# Tumor Cells Modified with Newcastle Disease Virus Expressing IL-24 as a Cancer Vaccine

**DOI:** 10.1016/j.omto.2019.06.001

**Published:** 2019-06-12

**Authors:** Xiaojing Xu, Cheng Yi, Xiaoqin Yang, Jianwei Xu, Qing Sun, Yonghao Liu, Lixiang Zhao

**Affiliations:** 1 College of Basic Medicine and Biological Sciences, Medical Department, Soochow University, 215123 Suzhou, China; 2 National Guizhou Joint Engineering Laboratory for Cell Engineering and Biomedicine Technique, Center for Tissue Engineering and Stem Cell Research, Guizhou Province Key Laboratory of Regenerative Medicine, Guizhou Medical University, 550004 Guiyang, Guizhou, China; 3 Laboratory of Adult Stem Cell Translational Research, Chinese Academy of Medical Sciences, Guiyang, Guizhou, China; 4 Laboratory of Animal Infectious Diseases, College of Veterinary Medicine, Yangzhou University, Yangzhou 225009, China; 5 Virus Research Unit, Department of Microbiology and Immunology, School of Medicine, University of Otago, Dunedin, New Zealand; 6 Institute of Blood and Marrow Transplantation, Department of Hematology, Collaborative Innovation Center of Hematology, the First Affiliated Hospital of Soochow University, 215123 Suzhou, People’s Republic of China

**Keywords:** interleukin-24, Newcastle disease virus, T cell, tumor, vaccine

## Abstract

Interleukin-24 (IL-24) is a promising agent for cancer immunotherapy that induces apoptosis of tumor cells and enhances T cell activation and function. In order to improve the antitumor activity induced by Newcastle disease virus (NDV)-modified tumor vaccine, we generated a recombinant NDV expressing IL-24 using reverse genetics. Irradiated tumor cells infected with LX/IL-24 showed stable IL-24 expression. The cytotoxicity assay showed that LX/IL-24-infected murine melanoma cells significantly enhanced the antitumor immune response *in vitro*. Then, the antitumor effects of virus-infected tumor cells were examined in the murine tumor models. LX/IL-24-infected tumor cells exhibited strong antitumor effects both in prophylaxis and therapeutic models. LX/IL-24-infected tumor cells increased infiltration of CD4^+^ T cells and CD8^+^ T cells in tumor sites, and the antitumor activity of the tumor vaccine modified with LX/IL-24 was dependent on CD8^+^ T cells. Taken together, our data well illustrates that LX/IL-24-modified tumor cells are a promising agent for cancer immunotherapy.

## Introduction

Newcastle disease virus (NDV) belongs to the genus *Avulavirus*.^1^ The genome of NDV contains six structural genes, including nucleoprotein (NP), phosphoprotein (P), matrix protein (M), fusion protein (F), hemagglutinin-neuraminidase (HN), and large polymerase protein (L).^2^, ^3^ NDV has been considered as an effective and safe agent for cancer therapy for more than 50 years.^4^, ^5^, ^6^ Like other oncolytic viruses, NDV has the ability to selectively replicate in tumor cells instead of normal cells,^7^, ^8^, ^9^ which makes it an effective agent for cancer immunotherapy.

NDV can be divided into two types (lytic and nonlytic) according to the pattern of infection in tumor cells. Lytic NDV produced infectious particles that could infect other tumor cells, while nonlytic NDV produced uninfectious particles.^10^, ^11^, ^12^ Lytic NDV is often used to lyse tumor cells directly, while nonlytic NDV is often used to modify tumor cells to use as vaccine (autologous tumor vaccines [ATVs] modified with NDV [ATV-NDVs]).^13^, ^14^ The therapeutic effects of ATV-NDV have been enhanced by insertion of immune-stimulating genes to NDV using reverse-genetic methods.

Interleukin-24 (IL-24), a novel cytokine, has multiple mechanisms of anti-tumor effects, such as inducing tumor cells apoptosis,^15^ promoting “bystander” effects,^16^ and enhancing T cell responses.^17^ IL-24 can also enhance immunogenicity of tumor cells by upregulating costimulatory molecules such as CD80 and CD86 on tumor cells.^18^, ^19^ Therefore, IL-24 may enhance therapeutic effects of ATV-NDV. NDV strain Laoxi (LX), a typical nonlytic virus, has been used to modified tumor cells as ATV-NDV for cancer immunotherapy. Several recombinant LX strains expressing therapeutic genes have been generated by reverse genetic system of LX and tested in our previous works.^14^, ^20^, ^21^ Here, we constructed a recombinant NDV strain carrying the IL-24 gene using reverse genetic system of nonlytic NDV LX. The antitumor effects of recombinant NDV expressing IL-24 were examined both *in vitro* and *in vivo.*

## Results

### Generation and Growth Curves of LX/IL-24

**Figure 1:**
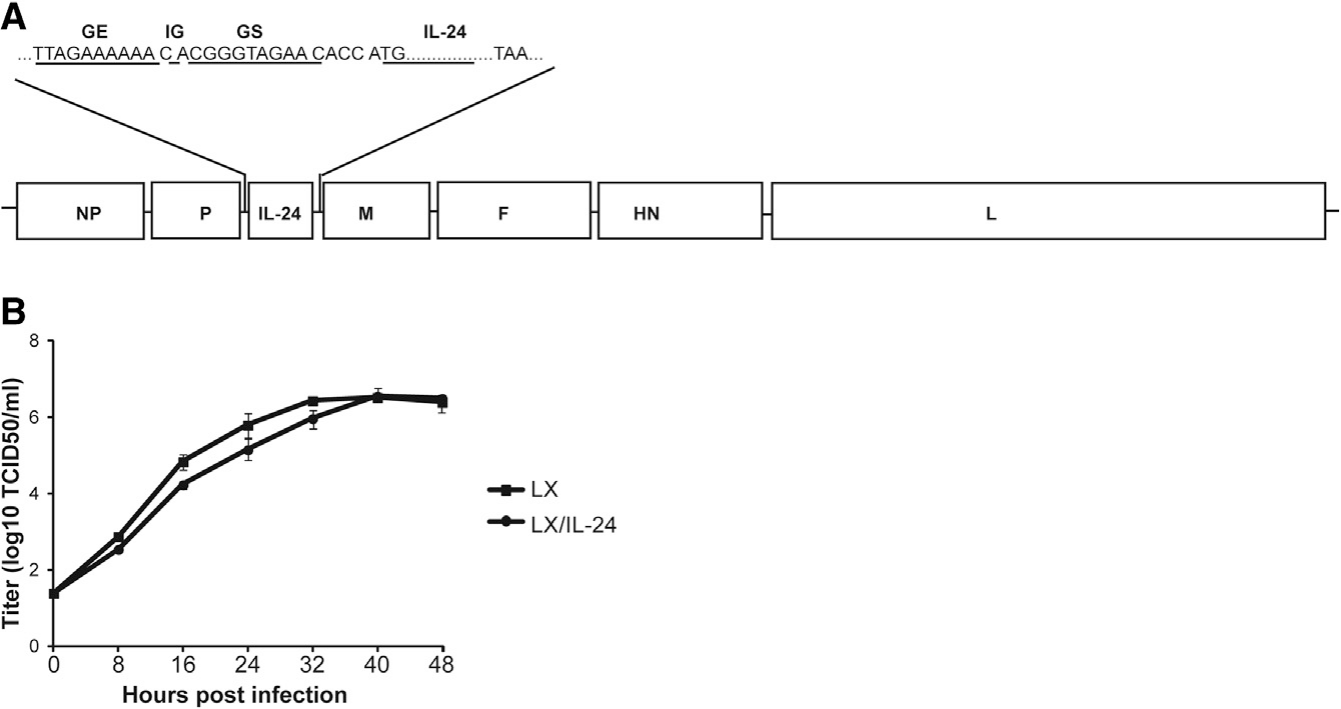
Construction of LX/IL-24 (A) Schematic representation of recombinant LX construct. The coding sequence IL-24 was inserted into the sequence between P and M genes of LX genome. (B) The virus titers were determined in triplicate by TCID50 in DF-1 cells at 0, 8, 16, 24, 32, 40, and 48 hr post-infection. The data shown are the representative of three experiments.

The gene encoding IL-24 was inserted into the region between P and M genes of LX genome according to the rule of six (Figure 1A). After co-transfection to BSR T7/5 cells with the support plasmids, recombinant NDV virus carrying IL-24 was obtained and termed LX/IL-24. Gene sequencing showed that insertion of IL-24 did not induce any mutation during virus construction.

The growth curves of LX and LX/IL-24 was determined in DF-1 cells, and each virus exhibited similar growth rate at the selected time points (Figure 1B). The highest titers each virus was at 40 hr post-inoculation.

### Expression of IL-24 in Tumor Cells Infected with Recombinant Virus

**Figure 2:**
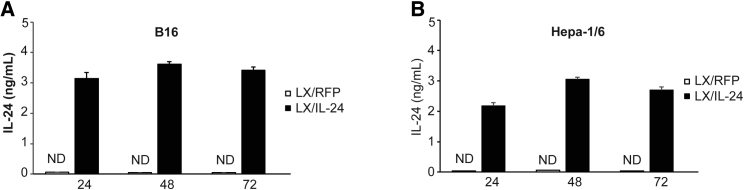
Expression IL-24 in Tumor Cells Infected with LX/IL-24 B16-F10 or Hepa-1/6 cells were irradiated with 150 Gy via a ^60^Go source and then incubated with LX/IL-24 or LX/RFP (100 HAU virus per 10^6^ cells). After incubation for 24, 48, and 72 hr, the supernatants from (A) B16 and (B) Hepa-1/6 were collected and detected for the production of IL-24 by ELISA. The data shown are the representative of three experiments. The significance levels are marked *p < 0.05 and **p < 0.01.

The expression of IL-24 in tumor cells infected by recombinant virus was determined using B16-F10 and Hepa-1/6 cells. LX/IL-24-infected tumor cells constantly expressed IL-24, while LX/RFP-infected tumor cells did not produce detectable IL-24. The highest amount of IL-24 productions were at 48 hr in tumor cells after LX/IL-24 infection (Figure 2).

### Killing Capacities of Splenocytes Stimulated by LX/IL-24-Infected Tumor Cells

**Figure 3:**
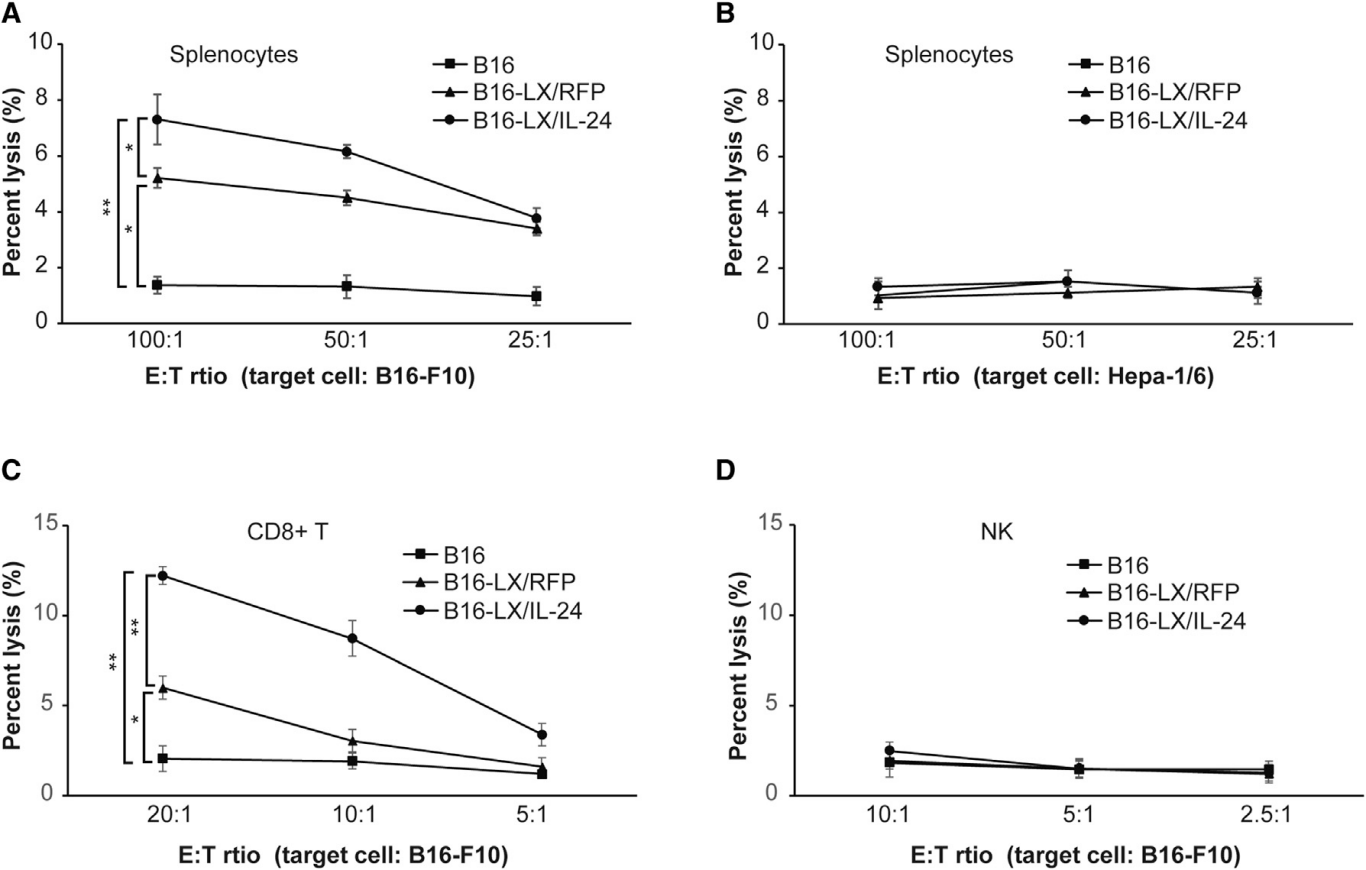
Killing Capacities of Splenocytes Stimulated with Virus-Infected Tumor Cells Splenocytes harvested from C57BL/6 mice were incubated with irradiated B16-F10 cells or irradiated B16-F10 cells infected with recombinant viruses for 6 days and used as effector cells. The killing capacity against B16-F10 (A) or Hepa-1/6 (B) cells was measured using CytoTox 96 nonradioactive cytotoxicity assay kit. CD8^+^ T cells (C) or NK cells (D) were sorted from above splenocytes and used as effector cells, and their killing capacities of B16-F10 targets were also measured. The data shown are the representative of three experiments. *p < 0.05 and **p < 0.01.

Antitumor effects induced by LX/IL-24-infected tumor cells were first examined *in vitro*. Splenocytes stimulated with LX/IL-24-infected B16-F10 exhibited significantly enhanced killing capacity against autologous tumor cells, as compared with irradiated B16-F10 or LX/RFP-infected B16-F10 stimulated splenocytes. LX/RFP-infected B16-F10 also enhanced killing capacities of splenocytes as tumor cells without virus infection (Figure 3A). No detectable cytotoxicity was observed when Hepa-1/6 cells were used as the targets, suggesting that the killing capacity of the splenocytes stimulated with LX/IL-24-infected tumor cells was tumor specific (Figure 3B).

To determine the effector cells responsible for the increased cytotoxicity, CD8^+^ T and NK cells were isolated from each group, respectively. CD8^+^ T cells from the LX/IL-24-infected tumor cell group exhibited significantly higher cytotoxicity against B16-F10 cells than other groups (Figure 3C), whereas NK cells from all groups showed very low cytotoxicity against B16-F10 targets (Figure 3D). These results demonstrated that LX/IL-24-modified tumor cells could promote the tumor-specific killing by CD8^+^ T cells.

### Prophylaxis with LX/IL-24 Infected Tumor Vaccine

**Figure 4:**
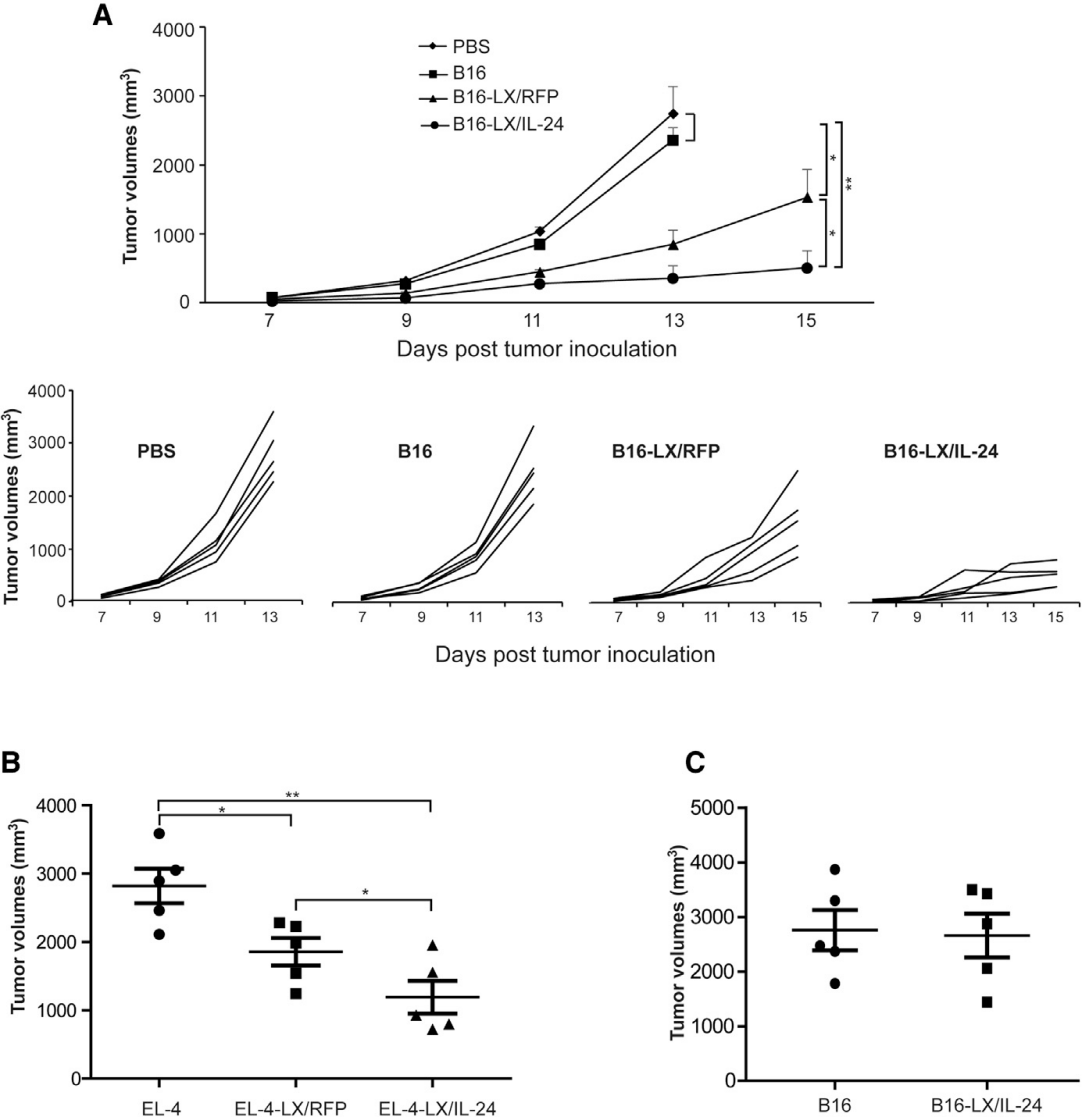
Prophylaxis Effect of LX/IL-24-Infected Tumor Cells (A) Mice were immunized with irradiated B16-F10, irradiated B16-F10 infected with LX/RFP, or irradiated B16-F10 infected with LX/IL-24 twice with 1-week intervals, respectively, then mice were challenged with 1 × 10^5^ B16-F10 cells. (B) Mice were immunized with irradiated EL-4, irradiated EL-4 infected with LX/RFP, or irradiated EL-4 infected with LX/IL-24 twice with 1-week intervals, respectively, then mice were challenged with 1 × 10^5^ EL-4 cells. (C) Mice were immunized with irradiated B16-F10 or irradiated B16-F10 infected with LX/IL-24, and challenged with EL-4 cells. The tumor volumes were monitored. The experiments were performed with five mice per group. *p < 0.05 and **p < 0.01.

To determine the *in vivo* protective effects of LX/IL-24-infected tumor vaccine, C57BL/6 mice were immunized with PBS, irradiated B16-F10 (B16), LX/RFP-infected B16-F10 cells (B16-LX/RFP), or LX/IL-24-infected B16-F10 cells (B16-LX/IL-24). Then, the immunized mice were challenged with B16-F10 cells. Mice immunized with B16-LX/IL-24 significantly inhibited tumor growth (Figure 4A). Immunization with B16-LX/RFP also inhibited tumor growth as compared with mice immunized with irradiated B16. Irradiated B16 cells have no obvious preventive effects against melanoma when compared with PBS group. The protective effect of LX/IL-24-modified tumor cells was also examined in murine lymphoma model (EL-4; Figure 4B). Immunization with LX/IL-24-modified tumor cells further reduced tumor growth compared with the immunization with LX/RFP-modified tumor cells. To determine whether the protective effects provided by LX/IL-24-modified tumor cell immunization were tumor specific, B16-LX/IL-24 immunized mice were also challenged with EL-4 cells. The results showed that B16-LX/IL-24 could not provide any increased preventive effects against EL-4 cells, as compared with irradiated B16-immunized mice (Figure 4C), suggesting that the antitumor response induced by LX/IL-24-modified tumor cells was specific to autologous tumor.

### Therapeutic Effects of LX/IL-24-Infected Tumor Vaccine

**Figure 5:**
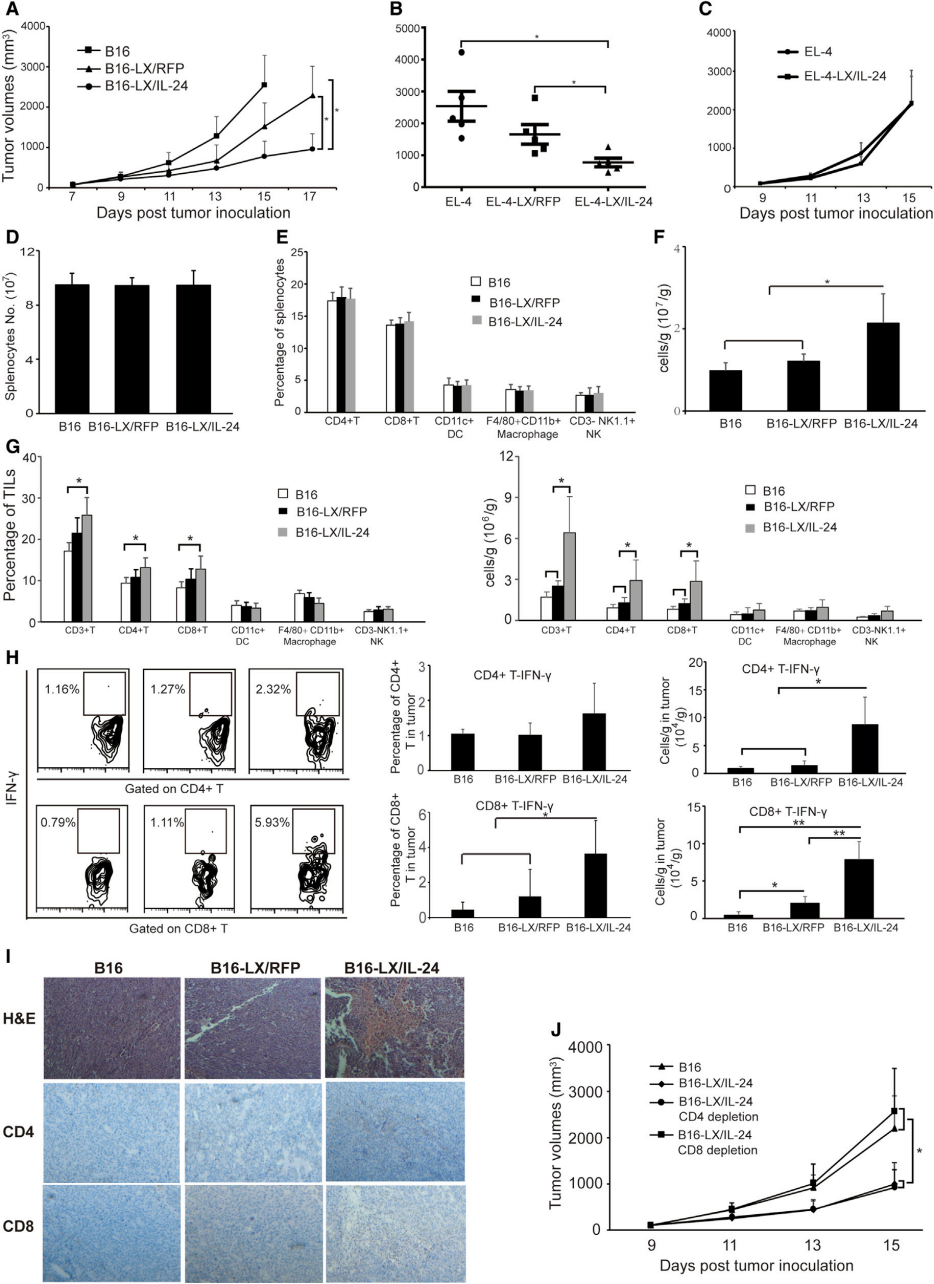
Therapeutic Effects of Tumor Vaccine Modified with LX/IL-24 (A) C57BL/6 mice were s.c. inoculated at the right flank with 5 × 10^4^ B16-F10 cells. On day 5, the left flank of the tumor-bearing animal was s.c. immunized with irradiated B16 cells, B16-LX/RFP, or B16-LX/IL-24. The inoculation of vaccines was repeated on day 9, and the tumor volumes were monitored. (B) C57BL/6 mice were s.c. inoculated at the right flank with 5 × 10^4^ EL-4 cells. Tumor-bearing mice were s.c. immunized with irradiated EL-4 cells, EL-4-LX/RFP, or EL-4-LX/IL-24 at day 5 and 9. The tumor volumes were monitored. (C) Melanoma-bearing mice were s.c. immunized with irradiated EL-4 cells or EL-4-LX/IL-24 at day 5 and 9. The tumor volumes were monitored. (D–I) Mice from melanoma model were sacrificed on day 15 post-tumor-inoculation. Total numbers of splenocytes (D) and frequencies of immune cells in the spleen (E) were examined. (F) Lymphocyte numbers per tumor weight were analyzed. Frequencies and numbers per tumor weight of tumor-infiltrating lymphocytes (TILs) (G) and IFN-γ production of CD4^+^ T and CD8^+^ T cells (H) were analyzed by FACS. (I) H&E and immunochemical staining for CD4^+^ and CD8^+^ T cells of tumor tissues from melanoma-bearing mice (200× magnification). (J) Mice received i.p. injections of 1 mg GK1.5 (rat anti-mouse CD4 mAb) or 500 μg 53-6.7 (rat anti-mouse CD8 mAb) 2 days before the first administration of B16-LX/IL-24, and the injections were repeated 7 days later. The tumor volumes were monitored daily. The experiments were performed with five mice per group. *p < 0.05 and **p < 0.01.

Therapeutic effects of LX/IL-24-infected tumor vaccine were furtherly determined in C57BL/6 mice. In the melanoma model, tumor-bearing mice were immunized with tumor vaccines on days 5 and 9, respectively. B16-LX/IL-24 immunization dramatically inhibited tumor growth, as compared with the B16-LX/RFP or B16 groups (Figure 5A). B16-LX/RFP only slightly inhibited tumor growth as compared with B16 group. The therapeutic effect of LX/IL-24 modified tumor cells was also confirmed in murine lymphoma model (EL-4; Figure 5B). To determine whether the therapeutic effects provided by LX/IL-24-modified tumor cell immunization were tumor specific, melanoma-bearing mice were also treated with irradiated EL-4 cells or irradiated EL-4 cells modified with LX/IL-24 (Figure 5C). EL-4-LX/IL-24 immunization cannot inhibit B16 melanoma growth as compared to the B16 group, suggesting that the therapeutic effect of LX/IL-24-modified tumor cells was specific to autologous tumor. Splenocytes and tumor-infiltrating lymphocytes (TILs) were prepared and examined by flow cytometry on day 15 after tumor inoculation. The percentages and numbers of CD4^+^ T, CD8^+^ T, dendritic cells, macrophages, and NK cells in spleen were similar from different treatment (Figures 5D and 5E). Absolute numbers of TILs per tumor weight were significantly increased in the B16-LX/IL-24 group, as compared with other groups (Figure 5F). The percentages and absolute numbers per tumor weight of tumor-infiltrating CD3^+^ T, CD3^+^ CD8^+^, and CD3^+^ CD4^+^ T cells were significantly enhanced after B16-LX/IL-24 immunization (Figure 5G), which suggested that LX/IL-24-modified tumor cells promoted antitumor responses by increased T cell infiltrations in the tumor. These results were also confirmed by H&E staining and immunohistochemistry staining (Figure 5I). Tumor-infiltrating T cell functions were determined *in vitro* by stimulation with B16-F10 cell lysates and intracellular staining of interferon-γ (IFN-γ). The percentages and absolute numbers per tumor weight of IFN-γ-producing CD8^+^ T cells were significantly enhanced in B16-LX/IL-24-treated group (Figure 5H). Although the percentages of IFN-γ-producing CD4^+^ T cells were slightly increased after B16-LX/IL-24 treatment, absolute numbers per tumor weight of these cells were significantly increased, compared with other groups.

To determine whether CD4^+^ or CD8^+^ T cells are critical for the antitumor activity induced by B16-LX/IL-24, CD4^+^ or CD8^+^ T cell depletion was performed in a therapeutic model (Figure 5J). Depletion of CD4^+^ did not change the therapeutic effects of B16-LX/IL-24, while depletion of CD8^+^ T cell depletion abrogated the antitumor effects of B16-LX/IL-24. These results indicate that the antitumor response of LX/IL-24-modified tumor cells was mediated by CD8^+^ T cells.

## Discussion

To promote antitumor responses induced by ATV-NDV, a recombinant NDV LX expressing IL-24 was generated and used to modify tumor vaccines. Then, the characteristics of recombinant virus and antitumor effects induced by virus-infected tumor cells were examined both *in vivo* and *in vitro*. Here, we found that antitumor effects were dramatically increased by tumor vaccine modified with NDV expressing IL-24.

After infection of tumor cells, NDV replicates in cytoplasm of host cells and never integrates its genome into the host’s, which makes it safer than other oncolytic viruses for immunotherapy.^22^, ^23^, ^24^ According to the pattern of infection in tumor cells of NDV, it can be divided into lytic and nonlytic strains. The lytic are used to lyse tumor cells directly, whereas the nonlytic are often used to generate whole-tumor-cell vaccines.^25^ ATV-NDVs are made from whole-cell tumor cells infected with nonlytic NDV, which were first developed by Schirrmacher and Heicappell in the 1980s.^26^ Clinic benefits of ATV-NDV were observed in many tumor types, such as glioblastoma multiforme, head and neck squamous carcinoma, renal cell cancer, and so on. LX strain, a typical nonlytic NDV, is a promising vector for the generation of ATV.^21^

T cells are critical effectors in antitumor immunity, and enhancement of T cell response is key to treating cancer.^27^, ^28^, ^29^ Recombinant NDV-expressing cytokines that can promote T cell responses will exhibit more powerful effects against cancer. IL-24 has multiple mechanisms of anti-tumor effects, including inducing tumor cell apoptosis, promoting “bystander” effects, and enhancing immunogenicity of tumor cells by upregulating costimulatory molecules that favor activation of T cells. Therefore, tumor cells modified with recombinant nonlytic NDV expressing IL-24 may have superior effects of stimulating T cell responses than those with parental virus. A previous study showed that tumor cells modified with a replication-deficient adenovirus expressing IL-24 can enhance the antitumor immune responses,^30^ while using nonlytic NDV as a vector to transfer IL-24 to tumor cells is superior to using replication-deficient adenovirus. First, NDV, a bird virus, cannot infect human normal cells, which makes it a safe vector for clinical use. On the contrary, tumor cells provide a relatively permissive substrate for NDV propagation, owing to mutations of the antiviral interferon system that makes them more susceptible to viral infections than normal cells.^22^ Second, HN proteins from NDV help expose tumor antigens and serve as costimulatory molecules of T cells to promote T cell responses. Third, nonlytic NDV modification will not lyse the tumor cells, and these whole-tumor-cell vaccines are more antigenic and can stimulate T cell responses more efficiently than oncolysates. Our study also showed that LX/RFP-infected tumor cells promoted antitumor responses both *in vitro* and *in vivo*, as compared with tumor cells without NDV infection, suggesting that LX is an effective vector for the construction of ATV-NDV in inducing antitumor immune responses.

The NDV genome is a nonsegmented, single-stranded, negative-sense RNA that encodes six structural proteins, NP-P-M-F-HN-L and NP, and the recombinant NDV should be generated by reverse genetic methods. We previously constructed the reverse genetic methods of nonlytic NDV strain LX and have successfully generated several recombinant LX viruses. The insertion sites of heterogeneous genes in the LX genome is critical for virus production and heterogeneous gene expression. In the present work, IL-24 were inserted between P and M, and the insertion did not affect the replication of recombinant virus, as compared with the parental strain. The recombinant virus also efficiently expressed IL-24 on tumor cells, which makes it a suitable vector for ATV-NDV generation.

*In vitro* and *in vivo* studies were performed to examine the antitumor effects of LX/IL-24-modified tumor cells. The *in vitro* study demonstrated that LX/IL-24 significantly enhanced tumor-specific cytotoxicity of splenocytes, as compared with parental virus. In preventive models, LX/IL-24 dramatically enhanced the antitumor immune response. Next, we examined the therapeutic effects of LX/IL-24-modified tumor vaccines, and significant inhibition of tumor growth was observed after being treated with LX/IL-24-modified tumor vaccines, indicating the production of IL-24 by NDV-infected vaccine cells may enhance antitumor effects.

Although percentages of most immune cells were not affected after different vaccine treatments, a significantly higher proportion and number of CD4^+^ and CD8^+^ T cells were observed in TILs after treatment with LX/IL-24-modified tumor vaccines. LX/IL-24-modified tumor vaccine treatment also enhanced the function of the CD8^+^ T cells, as indicated by significantly higher proportion and numbers of IFN-γ-producing CD8^+^ T cells. The increased T cell infiltration in the tumor microenvironment is often correlated with improved clinical outcome in several cancers.^31^, ^32^, ^33^, ^34^ Recent studies also suggested that antitumor effects of immunotherapy by NDV could be enhanced when combined with immune checkpoint antibodies.^35^, ^36^ Therefore, combining with immune checkpoint antibodies such as CTLA4 or PD-1 may further promote the therapeutic efficacy of LX/IL-24-modified tumor vaccines.

Thus, we generated a recombinant nonlytic NDV virus expressing IL-24, and the recombinant virus could greatly enhance the prophylaxis and therapeutic effects against cancer. It warrants future studies for the detailed mechanisms of the antitumor effects by this virus-modified tumor vaccine.

## Materials and Methods

### Animals

Specific-pathogen-free (SPF) 6-week-old female C57BL/6 mice were purchased from SLC (Shanghai, China). Animal care and experimental procedures were performed under SPF conditions. All animal protocols were approved by the Institutional Laboratory Animal Care and Use Committee at Soochow University (permit number: 201809A462). At the end of the experiments, animals were euthanized in a CO_2_-containing chamber.

### Cells and Viruses

Murine melanoma cell line (B16-F10), murine hepatocarcinoma cell line (Hepa-1/6), murine T-lymphoma cell line (EL-4), and chicken fibroblast cell line (DF-1) obtained from the American Type Culture Collection were cultured in complete DMEM medium (Gibco) with 10% fetal bovine serum (FBS). Mycoplasma testing of each cell line was negative. Recombinant NDV LX strain carrying red fluorescent protein (LX/RFP) and recombinant LX strain carrying IL-24 (LX/IL-24) were grown in 10-day-old embryonated SPF eggs.

### Generation of Recombinant LX Carrying IL-24

DNA fragments of IL-24 flanking by NDV gene end (GE), intergenic (IG), and gene start (GS) sequences were inserted between the P and M gene of LX antigenomic cDNA (Figure 1). After cotransfection of this plasmid to BSR T7/5 cells with support plasmids (encoding N, P, and L genes) from ZJ1 strain, the recombinant virus LX/IL-24 was generated. Then, transfected BSR T7/5 cell supernatant was inoculated into allantoic cavities of 10-day-old embryonated SPF eggs, and the virus from allantoic fluid was screened by hemagglutination assay (HA) using 0.5% chicken red blood cells. The recombinant viruses were also examined by gene sequencing.

### Growth Kinetics of the Virus

The growth curves of the recombinant viruses were determined by a multistep growth assay in DF-1 cells as previously reported. In brief, DF-1 cells were infected with virus at 37°C in Eagle’s minimal essential medium (EMEM) supplemented with 10% uninfected egg allantoic fluid as a source of exogenous protease in a 5% CO_2_ atmosphere. Cells were collected and frozen at 0, 8, 16, 24, 32, 40, and 48 hr post-infection. 100 μL of 10-fold serial dilutions in PBS were added to plates containing 5 × 10^4^ DF-1/well. The titers of virus were determined by end-point titration and expressed as mean log_10_ 50% tissue culture infective dose (TCID50) per mL.

### Expression of IL-24 in Virus-Infected Tumor Cells

1 × 10^6^ B16-F10 or Hepa-1/6 cells were irradiated with 150 Gy via a ^60^Go source and co-cultured with 100 hemagglutination units (HAU) LX at 37°C, and uninfected viruses were removed by replacing supernatant with equal volume of fresh medium 1 h later. The supernatants were collected 24, 48, and 72 hr later, and amounts of IL-24 were detected by ELISA using mouse IL-24 DuoSet ELISA (R&D Systems, Minneapolis, MN, USA).

### Tumor Vaccine Production

1 × 10^6^ tumor cells were irradiated with 150 Gy via a ^60^Go source and co-cultured with 100 HAU NDV viruses for 1 hr. Uninfected viruses were removed, and the virus-containing cells were used as vaccine.

### Killing Assays

Killing capacities of splenocytes (effector) were measured using a CytoTox 96 nonradioactive cytotoxicity assay (Promega, Madison, WI, USA). In brief, splenocytes were diluted and cultured with targets (5,500 B16 or 6,500 Hepa-1/6) in a 96-well plate at effector/target ratios of 100:1, 50:1, and 25:1 at 37°C in a 5% CO_2_. The percentage of lysis was calculated using the following formula: (experimental − effector spontaneous − target spontaneous) × 100/(target maximum − target spontaneous).

### Animal Models

In prophylactic models, mice were immunized subcutaneously (s.c.) at the left flank with PBS, 1 × 10^6^ irradiated tumor cells, 1 × 10^6^ irradiated tumor cells infected with LX/RFP, or 1 × 10^6^ irradiated tumor cells infected with LX/IL-24 in a total volume of 100 μL, and the injections were repeated 1 week later. Two weeks after the last immunization, 1 × 10^5^ B16-F10 or EL-4 cells were s.c. implanted into the right flank of mice. Tumor volumes of melanoma were daily monitored. The mice from the EL-4 model were sacrificed on day 18 post-tumor-inoculation, and the tumor volumes were measured using the formula V = (L × W^2^)/2, where L is the length (longest dimension) and W is the width (shortest dimension). The experiments were performed with five mice per group.

In therapeutic experiments, five mice were s.c. inoculated with 5 × 10^4^ B16-F10 or EL-4 cells and s.c. immunized with tumor vaccines 5 and 9 days later, respectively. The tumor sizes of melanoma were daily monitored. The mice from the EL-4 model were sacrificed on day 17 post-tumor-inoculation, and the tumor volumes were measured. The experiments were performed with five mice per group.

### Depletion of CD4^+^ or CD8^+^ Cells

To deplete CD4^+^ or CD8^+^ cells *in vivo*, 1 mg GK1.5 (rat anti-mouse CD4 monoclonal antibody [mAb]) and 500 μg 53-6.7 (rat anti-mouse CD8 mAb) were intraperitoneally (i.p.) injected to each mouse 2 days before the first immunization, and the injections were repeated 7 days later, respectively. The experiments were performed with five mice per group.

### Flow Cytometry

Spleens and tumors were harvested from mice, and lymphocytes were isolated as previously described.^20^ As for surface staining, cells were blocked with CD16/32 FcR-block (BioLegend, San Diego, CA, USA) for 15 min and stained with fluorescent dye-conjugated mAb (BD Biosciences, Franklin Lakes, NJ, USA) for 30 min at 4°C. For intracellular cytokine staining, cells were stimulated with B16-F10 for 18 h in the presence of brefeldin A (10 μg/mL; BD Biosciences, San Jose, CA, USA). Cells were stained for surface markers for 30 min, then fixed with 4% paraformaldehyde, permeabilized with 1% saponin (Sigma-Aldrich, St. Louis, MO, USA), and stained for cytokines for 30 min at 4°C. The antibodies used for fluorescence-activated cell sorting (FACS) staining were as follows: fluorescein isothiocyanate (FITC)-conjugated CD4 (RM4-5), FITC-conjugated NK1.1 (PK136), phycoerythrin (PE)-conjugated anti-CD3ε (145-2C11), peridinin-chlorophyll-protein complex (PerCP) Cy5.5-conjugated Gr-1 (RB6-8C5), allophycocyanin-conjugated CD11b (M1/70), PE-Cy-conjugated CD11c (N418), FITC-conjugated F4/80 (T45-2342), allophycocyanin (APC)-conjugated IFN-γ (XMG1.2), and PerCP Cy5.5-conjugated CD8a (53-6.7). All staining was performed in FACS buffer (1× PBS, 1% BSA, and 0.1% NaN_3_). The flow cytometric results were analyzed with FACS Calibur (BD Biosciences, San Jose, CA, USA) using CellQuest software.

### Immunohistochemistry and Histopathology

Tumor tissues from tumor-bearing mice were fixed in 4% formalin at room temperature for 2 days, processed through graded concentrations of ethanol and xylene, and embedded in paraffin wax. The sections of 4–5 mm were mounted on adhesive glass slides and stained with H&E. Then the sections were deparaffinized and treated with 0.08% H_2_O_2_ for 30 min to block endogenous peroxidase. Slides were incubated with rat anti-CD4 (GK1.5; Abcam, Cambridge, MA, USA) or rat anti-CD8 (2.43; Abcam, Cambridge, MA, USA) at 4°C overnight and incubated with horseradish peroxidase (HRP)-conjugated rabbit anti-rat immunoglobulin (Ig). Diaminobenzidine was used to develop the staining reaction, and nuclear counterstaining was performed with hematoxylin. Slides were coded and examined by a pathologist who was blinded for the experimental history of the animals.

### Statistical Analysis

All data were analyzed by Student’s t test and expressed as means ± SEM. Data were analyzed using GraphPad Prism 5 software for Windows (GraphPad Software, San Diego, CA, USA), and differences were considered statistically significant when p < 0.05. The significance levels are marked *p < 0.05 and **p < 0.01.

## Author Contributions

X.X. and C.Y. conducted the experiments. L.Z. and X.Y. designed the experiments and wrote the paper. Y.L. , Q.S., and J.X. analyzed and interpreted of data.

## Conflicts of Interest

The authors declare no competing interests.
